# SOCIAL AND BIOLOGICAL PREDICTORS OF HOSPITAL ADMISSIONS FOR A FALL IN THE ENGLISH LONGITUDINAL STUDY OF AGEING (ELSA)

**DOI:** 10.1093/geroni/igz038.233

**Published:** 2019-11-08

**Authors:** Jessica G Abell, Camille Lassale, Andrew Steptoe, G David Batty, Paola Zaninotto

**Affiliations:** 1 University College London, London, United Kingdom; 2 IMIM (Hospital del Mar Medical Research Institute), Barcelona, Spain

## Abstract

Falls are the most frequent type of accidents among older people, with one in three people aged over 65 falling each year. Falls serious enough to result in hospital admission are especially problematic, since they can lead to an increased likelihood of future disability, loss of independence, and premature mortality. Understanding the factors that may determine the risk of experiencing a fall, which requires admission to hospital, is therefore an important priority. This paper seeks to examine this issue using Hospital Episode Statistics (HES) data – administrative data from English hospitals in the National Health Service (NHS). These data have recently been linked with the English Longitudinal Study of Ageing (ELSA). We examine the association between a range of predictors (demographic, social environment, physical and mental functioning) drawn from wave 4 of ELSA with the first occurrence of hospitalisation due to an accidental fall, identified using ICD-10 codes. Analysis using Cox regression suggest a range of factors are negatively associated with admission to hospital with diagnosis of a fall, such as living alone (HR=1.42; 95% CI: 1.19, 1.68), urinary incontinence (HR=1.33; 95% CI: 1.09, 1.61) and depressive symptoms (HR=1.50; 95% CI: 1.23, 1.82). High walking speed (HR=0.30; 95% CI: 0.23, 0.39) and good hand-grip strength (HR=0.97; 95% CI: 0.96, 0.98) were found to be protective. The prevention of serious falls amongst older people will require determinants to be identified and managed effectively by health and social care services.

